# The prognostic role of stress echocardiography in a contemporary population and the clinical significance of limited apical ischaemia

**DOI:** 10.1530/ERP-16-0033

**Published:** 2016-12

**Authors:** Alexandros Papachristidis, Damian Roper, Daniela Cassar Demarco, Ioannis Tsironis, Michael Papitsas, Jonathan Byrne, Khaled Alfakih, Mark J Monaghan

**Affiliations:** 1 Department of Cardiology, King’s College Hospital NHS Foundation Trust, London, United Kingdom; 2 Lewisham Healthcare NHS Trust, London, United Kingdom

**Keywords:** stress echocardiography, outcome, MACCE, apical ischaemia

## Abstract

**Introduction:**

In this study, we aim to reassess the prognostic value of stress echocardiography (SE) in a contemporary population and to evaluate the clinical significance of limited apical ischaemia, which has not been previously studied.

**Methods:**

We included 880 patients who underwent SE. Follow-up data with regards to MACCE (cardiac death, myocardial infarction, any repeat revascularisation and cerebrovascular accident) were collected over 12 months after the SE. Mortality data were recorded over 27.02 ± 4.6 months (5.5–34.2 months). We sought to investigate the predictors of MACCE and all-cause mortality.

**Results:**

In a multivariable analysis, only the positive result of SE was predictive of MACCE (HR, 3.71; *P* = 0.012). The positive SE group was divided into 2 subgroups: (a) inducible ischaemia limited to the apical segments (‘apical ischaemia’) and (b) ischaemia in any other segments with or without apical involvement (‘other positive’). The subgroup of patients with apical ischaemia had a significantly worse outcome compared to the patients with a negative SE (HR, 3.68; *P* = 0.041) but a similar outcome to the ‘other positive’ subgroup. However, when investigated with invasive coronary angiography, the prevalence of coronary artery disease (CAD) and their rate of revascularisation was considerably lower. Only age (HR, 1.07; *P* < 0.001) was correlated with all-cause mortality.

**Conclusion:**

SE remains a strong predictor of patients’ outcome in a contemporary population. A positive SE result was the only predictor of 12-month MACCE. The subgroup of patients with limited apical ischaemia have similar outcome to patients with ischaemia in other segments despite a lower prevalence of CAD and a lower revascularisation rate.

## Introduction

Stress echocardiography (SE) is an established and widely used imaging functional test. It is included in most guidelines for the investigation of chest pain (1). It is also used in the risk stratification of patients with known coronary artery disease (CAD) (2), valvular heart disease (3), pre-operative assessment (4) and in the assessment of myocardial viability (5). The guidelines have recommended the use of imaging functional tests in the diagnosis of intermediate-risk patients with suspected CAD (1), and SE has the advantage of being widely available, low cost, safe and a bed side technique (6).

The diagnostic efficacy of SE in the diagnosis of CAD has been demonstrated in previous studies (7, 8, 9). SE was also demonstrated to be a useful marker of prognosis in case series from the 1980s and 1990s (10, 11, 12). As the pathways for the investigation of chest pain are evolving, and the populations under investigation have a lower incidence of CAD (13), we wanted to evaluate the utility of SE in a contemporary setting, in a high-volume tertiary centre. We also assess the clinical significance of limited apical ischaemia and its correlation with angiographic findings.

## Methods

927 consecutive patients who underwent SE (dobutamine or exercise) between 01/01/2012 and 31/12/2012 at a UK tertiary referral cardiac centre were included in the registry.

The dobutamine SE protocol used 3 min stages with incremental dobutamine doses of 5, 10, 20, 30 and 40 µg/kg/min. The test was terminated when either a new regional wall motion abnormality in 2 or more segments was detected or 85% of maximal predicted heart rate (HR) was reached. Bolus doses of intravenous atropine were given up to a maximum dose of 1200 µg if target HR was not achieved on dobutamine alone. Standard apical and parasternal images were acquired at baseline, low dose, intermediate dose and at peak heart rates. The exercise SE used a Bruce treadmill exercise protocol, aiming to reach the maximum predicted heart rate. The standard images were acquired at rest and immediately after peak exercise. The test was regarded as being sub-maximal if 85% of the maximum predicted heart rate was not achieved. Ultrasound contrast agent was administered if more than 2 myocardial segments were not visible on the baseline images. Beta-blockers were withheld for 2 days prior to the test.

The SE images were reviewed by an experienced Imaging Consultant and were considered positive if at least 2 adjacent segments in the same vascular territory (14) demonstrated evidence of ischaemia (deteriorating function or biphasic response). We performed a further classification of all positive SE as follows:

- ‘Limited apical’ if inducible ischaemia was demons­trated in two or more of the 4 apical segments only.
- ‘Other positive’ if ischaemia was demonstrated in other segments with or without the involvement of apical segments.

The EF at rest was calculated using the Simpson’s biplane method.

The demographic data (age and sex), cardiovascular risk factors (hypertension, hypercholesterolaemia, diabetes, family history, smoking status and history of CAD), the indication for SE and the result of the test were entered prospectively during the SE. Angiographic data were collected retrospectively from hospital’s records. All invasive angiograms were reported by an experienced interventional cardiologist. A 50–75% narrowing in the lumen of a coronary artery was considered a moderate stenosis. A narrowing below 50% and greater than 75% was considered mild and severe stenosis, respectively. The decision for coronary angiography was made by the clinician who was in charge for each patient’s management and was based on his individual judgement and patient’s preference.

Follow-up and outcome data were collected from electronic patient records. Mortality data were obtained from the National Health Care Records Service database. Major Adverse Cardiac and Cerebrovascular Events (MACCE) were defined as cardiac death, myocardial infarction, any repeat revascularisation for acute coronary syndrome and cerebrovascular accident. We collected data with regards to MACCE for a fixed follow-up period of 12 months after the SE. We also recorded all-cause mortality data over a different period of 27.0 ± 4.6 months (5.5–34.2 months).

All data were analysed with IBM SPSS, version 20.0.0 (IBM Corporation Software Group) and STATA, version 12.0 (Stata Corp, College Station, Texas, USA). The normality of the distribution for continuous variables was tested using the Shapiro–Wilk test. The Student’s *t*-test and the non-parametric Mann–Whitney *U* test were used as appropriate to compare mean values of continuous variables, and the values are presented as a mean value ± s.d. Categorical variables were tested with chi-square test and Fisher’s exact test as appropriate. Variables were tested with relation to MACCE using a Cox regression model. All tests of significance were two tailed. *P* values ≤0.05 was the criterion used to determine statistical significance. The proportionality of hazards assumption was assessed using the Schoenfeld test, and the assumption was met.

This study is not classified as research under NHS Health Research Authority Guidance, so we did not need to apply for Ethical Approval or patient permission. The study was assessed as a service evaluation by King’s College Hospital’s Audit Committee and the relevant permission was granted.

## Results

From the 927 patients, 9 patients had SE for viability assessment only. Cardiovascular risk factors were not recorded for 11 patients. In 27 patients, the target heart rate was not achieved, and these were considered non-diagnostic and were excluded from the analysis. The baseline characteristics of the 880 remaining patients are shown in Table 1. The age of the tested population was 61.0 ± 12.3 years and 50.0% were male. 210 (23.9%) tests were exercise SEs and 770 (76.1%) were dobutamine SEs. The average achieved METS was 10.3 ± 2.8 and the exercise time was 9.02 ± 2.68 min. There were 367 positive tests (41.7%) and 513 were negative (58.3%). Patients who had positive SE (Table 2) were significantly older, predominantly male and had higher prevalence of diabetes, hypertension and previous history of CAD. As expected, they were much more likely to be treated with revascularisation. Also, the use of ultrasound contrast agent was used more frequently in patients with positive SE. Contrast agent was also used more frequently in dobutamine SE (76.8%) compared to exercise SE (5.6%) tests (*P* < 0.001). The reason for this is our department’s policy to list patients for DSE in case of poor imaging window. We investigated both all-cause mortality outcome and MACCE. In the extended follow-up period of all-cause mortality, 30 patients died. We used a different follow-up period (a fixed period of 12 months from the date of the test) to record MACCE. The time of the first event was included in the analysis. In this period, 24 events were recorded. These 24 events include four patients who died of cardiac causes, 8 patients who suffered acute myocardial infarction with subsequent revascularisation, 7 patients who had late revascularisation for acute coronary syndrome/unstable angina and 5 patients who suffered a cerebrovascular accident.

**Table 1:** Baseline characteristics.

	**All patients** (*N* = 880)
Age	61.0 ± 12.3
Gender (male)	440 (50.0%)
Diabetes	225 (25.6%)
Smoking	109 (12.4%)
Hyperlipidaemia	524 (59.5%)
Hypertension	561 (63.8%)
Baseline EF	
Normal	750 (85.2%)
Mildly impaired	83 (9.4%)
Moderately/severely impaired	47 (5.3%)
Known CAD	286 (32.5%)
Positive FH	199 (22.6%)
US contrast agent	524 (59.5%)
Exercise stress echo	210 (23.9%)
Revascularisation	80 (9.1%)
Cardiac death	7 (0.8%)
MACCE	24 (2.8%)
All-cause mortality	30 (3.4%)

CAD, coronary artery disease; EF, ejection fraction; FH, family history for early coronary artery disease; MACCE, major adverse cerebral and cardiovascular events; US, ultrasound.

**Table 2:** Comparison of baseline characteristics between patients with negative and positive stress echo tests.

	**Test result**		
	**Negative 513** (58.3%)	**Positive 367** (41.7%)	***P* value**
Age	59.4 ± 12.8	63.3 ± 11.2	**<0.001**
Gender (male)	233 (45.4%)	207 (56.4%)	**0.001**
Diabetes	116 (22.6%)	109 (29.7%)	**0.017**
Smoking	61 (11.9%)	48 (13.1%)	0.598
Hyperlipidaemia	295 (57.5%)	229 (62.4%)	0.145
Hypertension	302 (58.9%)	259 (70.6%)	**<0.001**
Baseline EF			**<0.001**
Normal	467 (91.0%)	283 (77.1%)	
Mildly impaired	29 (5.7%)	54 (14.7%)	
Moderately/severely impaired	17 (3.3%)	30 (8.2%)	
Known CAD	116 (22.6%)	170 (46.3%)	**<0.001**
Positive FH	119 (23.2%)	80 (21.8%)	0.625
US contrast agent	218 (42.5%)	306 (83.4%)	**<0.001**
Revascularisation	4 (0.8%)	76 (20.7%)	**<0.001**
Cardiac death	1 (0.2%)	6 (0.7%)	**0.018**
MACCE	5 (1.0%)	19 (5.3%)	**<0.001**
All-cause mortality	13 (2.5%)	17 (4.6%)	0.091

CAD, coronary artery disease; EF, ejection fraction; FH, family history for early coronary artery disease; MACCE, major adverse cerebral and cardiovascular events; US, ultrasound. Bold values indicate statistical significance, *P* < 0.05.

**Figure 1:**
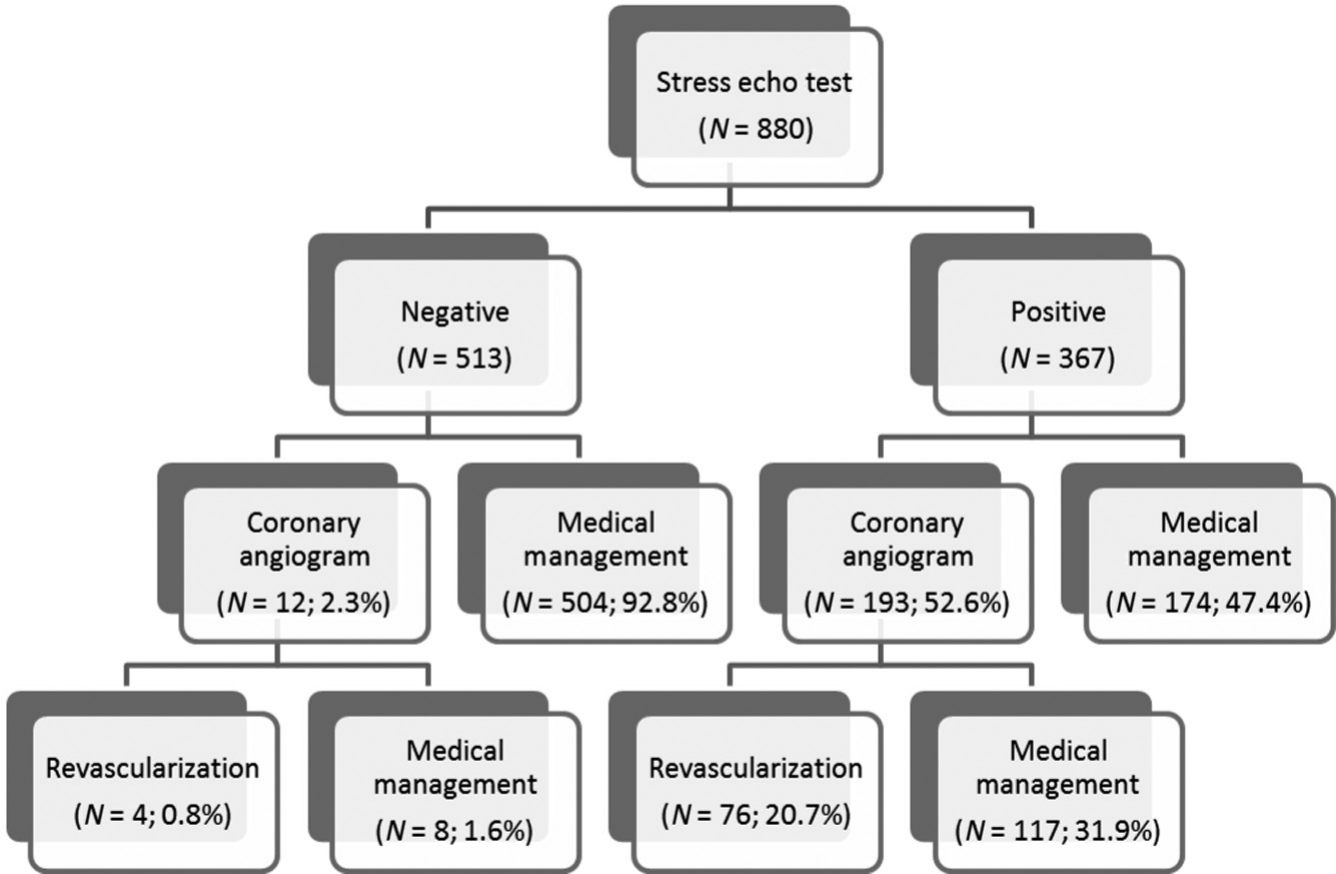
Flowchart showing the management of patients after the stress echocardiogram test.

The management of the patients after the SE test is shown in Fig. 1. From 367 patients who had a positive SE, 193 (52.6%) underwent invasive coronary angiography (ICA) and finally 76 (20.7%) were revascularised. 62 patients had percutaneous coronary intervention (PCI) and 14 were treated with coronary artery bypass grafting (CABG). From the 513 patients who had a negative SE test, only 12 patients (2.3%) had ICA and 4 (0.8%) were revascularised with PCI. Three patients had computed tomography coronary angiogram (CTCA) before the SE, and none had CTCA after the SE.

**Figure 2:**
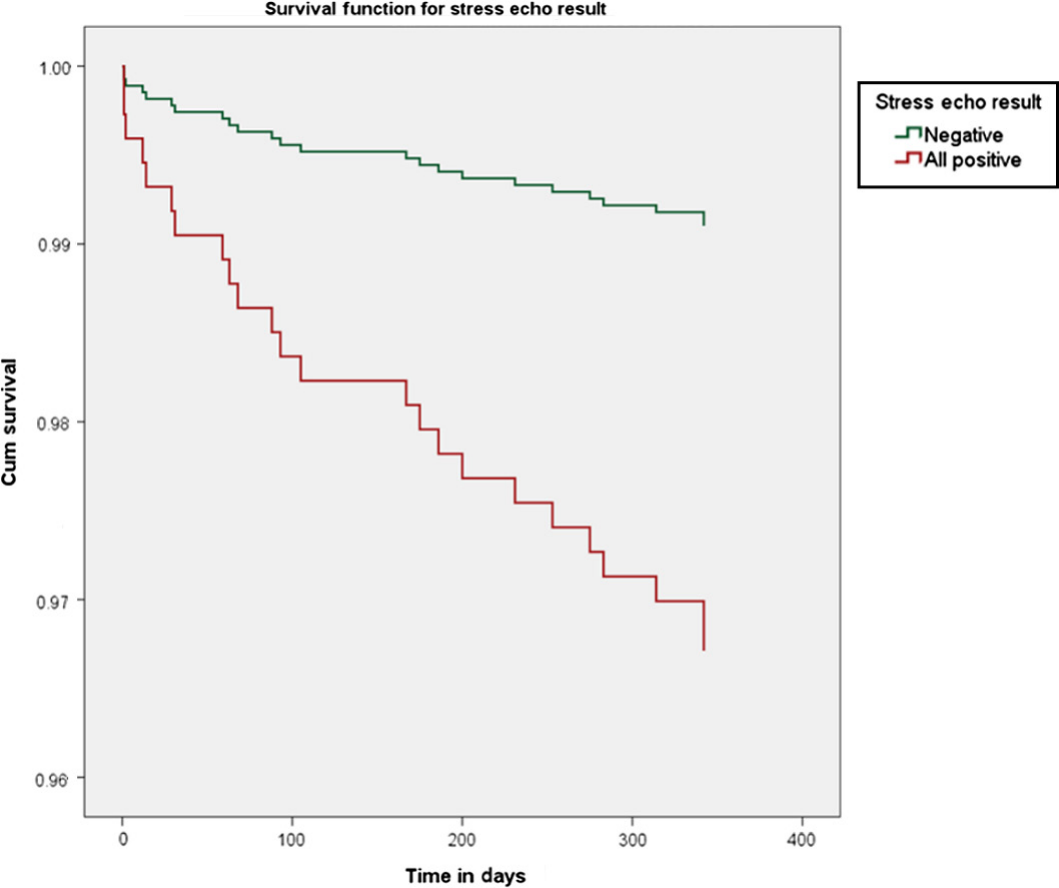
Cox regression survival curves for MACCE according to stress echo result (*P* = 0.012).

Initially, we sought to identify factors that could be predictors of MACCE. Ten variables (age, gender, diabetes, smoking, hyperlipidaemia, hypertension, history of CAD, family history of premature CAD, baseline EF and the result of the SE test) were investigated using univariable Cox regression analysis. It was not possible to retrieve follow-up data for 16 patients, and these were excluded from the analysis. Gender, history of CAD, EF and the positive SE result were found to be significantly related to MACCE (Table 3). Those variables along with diabetes and hypertension, which were related to MACCE at a level close to significance (*P* < 0.2), were tested in a multivariable Cox regression model with enter method. In the multivariable analysis, only the positive SE result was a predictor of MACCE in the follow-up period of 12 months (HR: 3.71; 95% CI: 1.34–10.27; *P* = 0.012). Cox regression survival curves for the SE result were calculated and are shown in Fig. 2.

**Table 3:** Univariable and multi-variable cox-regression analysis of predictors of MACCE (major adverse cerebral and cardiovascular events).

	**Univariable analysis**			**Multivariable analysis**		
**Variable**	**HR**	**CI**	***P***	**HR**	**CI**	***P***
Age	1.01	0.98–1.04	0.569			
Gender (male)	3.86	1.14–10.35	**0.007**	2.32	0.81–6.60	0.116
Diabetes	1.72	0.75–3.93	**0.198**	1.44	0.63–3.33	0.387
Smoking	1.41	0.48–4.13	0.528			
Hyperlipidaemia	1.33	0.57–3.11	0.509			
Hypertension	2.15	0.80–5.75	**0.128**	1.41	0.52–3.86	0.499
Known CAD	4.29	1.84–10.02	**0.001**	1.97	0.74–5.24	0.175
Positive FH	0.48	0.14–1.61	0.233			
Baseline EF			**0.009**			0.643
Normal (reference)	1.0	Reference			Reference	
Mildly impaired	3.12	1.13–8.59	**0.028**	1.39	0.48–4.05	0.549
Moderately/severely impaired	4.28	1.42–12.90	**0.010**	1.70	0.52–5.53	0.380
SE result						
Negative		Reference			Reference	
Positive	5.55	2.07–14.87	**0.001**	3.71	1.34–10.27	**0.012**

CAD, coronary artery disease; EF, ejection fraction; FH, family history for early coronary artery disease; SE, stress echocardiography. Bold values indicate statistical significance, *P *< 0.05.

We also compared the group of patients with positive SE who underwent invasive angiography to the group of patients with positive SE who did not have angiography. There was no difference in MACCE (13 patients; 6.7% vs 6 patients; 3.7%, *P* = 0.201). Also in a univariable Cox regression analysis, the invasive angiography after a positive SE test was not found to be a predictor of MACCE (HR: 1.89, 95% CI: 0.72–4.98; *P* = 0.196).

Subsequently, we investigated the same variables with relation to all-cause mortality (Table 4). In univariable analysis, age, gender, hypertension and baseline EF were related to mortality. The stress echo result was close to significant correlation with mortality (*P* < 0.2). These 5 variables were included in a Cox regression multivariable model and only age (HR: 1.07, 95% CI: 1.04–1.11; *P* < 0.001) was a predictor of mortality. Male gender was very close to statistically significant correlation with mortality (HR: 2.29; 95% CI: 1.00–5.26; *P* = 0.051). The positive result of the SE test was not related to mortality outcome (*P* = 0.483).

**Table 4:** Univariable and multi-variable cox-regression analysis of all-cause mortality.

	**Univariable analysis**			**Multivariable analysis**		
**Variable**	**HR**	**CI**	***P***	**HR**	**CI**	***P***
Age	1.08	1.04–1.12	**<0.001**	1.07	1.04–1.11	**<0.001**
Gender (male)	2.80	1.25–6.29	**0.013**	2.29	1.00–5.26	0.051
Diabetes	1.05	0.47–2.35	0.913			
Smoking	1.43	0.55–3.73	0.467			
Hyperlipidaemia	1.34	0.63–2.87	0.448			
Hypertension	2.85	1.09–7.44	**0.033**	1.63	0.61–4.37	0.331
Known CAD	1.21	0.58–2.55	0.611			
Positive FH	0.84	0.34–2.05	0.701			
Baseline EF			**0.005**			0.115
Normal (reference)	1.0	Reference			Reference	
Mildly impaired	3.46	1.45–8.23	**0.005**	2.43	1.00–5.93	0.051
Moderately/severely impaired	3.46	1.18–10.16	**0.024**	1.97	0.65–5.98	0.230
SE result						
Negative		Reference			Reference	
Positive	1.84	0.89–3.79	**0.098**	1.16	0.55–2.45	0.696

CAD, coronary artery disease; EF, ejection fraction; FH, family history for early coronary artery disease; SE, stress echocardiography. Bold values indicate statistical significance, *P *< 0.05.

We performed a subgroup analysis for the patients with a positive SE who were divided into a group of limited apical ischaemia and those with inducible ischaemia in other segments as described previously. The baseline characteristics of those subgroups are shown in Table 5. The patients with limited apical ischaemia were predominantly female, had a lower incidence of previous history of CAD and had better LV systolic function at rest. From the 145 patients with apical ischaemia, 6 (4.1%) had mild LV systolic dysfunction and only one (0.7%) had moderate LV systolic dysfunction. These patients were less frequently managed with ICA and revascularisation (*P* < 0.001). All-cause mortality was lower in the group of limited apical ischaemia. Cardiac death and MACCE did not differ significantly between the two groups, and this may be attributed to small number of events.

**Table 5:** Characteristics of patients with limited apical ischaemia vs those with inducible ischaemia in other segments (other positive).

	**Apical ischaemia** (*N* = 145)	**Other positive** (*N* = 222)	***P* value**
Age	62.7 ± 10.3	63.6 ± 11.7	0.339
Gender (male)	63 (43.4%)	144 (64.9%)	**<0.001**
Diabetes	41 (28.3%)	68 (30.6%)	0.629
Smoking	19 (13.1%)	29 (13.1%)	0.991
Hyperlipidaemia	90 (62.1%)	139 (62.6%)	0.916
Hypertension	95 (65.5%)	164 (73.9%)	0.086
CAD	36 (24.8%)	134 (60.4%)	**<0.001**
Positive FH	29 (20.0%)	51 (23.0%)	0.500
Coronary angiogram	58 (40.8%)	153 (72.9%)	**<0.001**
Revascularisation	12 (8.3%)	64 (28.8%)	**<0.001**
Baseline EF			**<0.001**
Normal	138 (95.2%)	145 (65.3%)	
Mildly impaired	6 (4.1%)	48 (21.6%)	
Moderately/severely impaired	1 (0.7%)	29 (13.1%)	
Cardiac deaths	1 (0.7%)	5 (2.3%)	0.409
MACCE	5 (3.5%)	14 (6.5%)	0.214
All-cause mortality	2 (1.4%)	15 (6.8%)	**0.017**

CAD, coronary artery disease; EF, ejection fraction; FH, family history for early coronary artery disease; MACCE, major adverse cerebral and cardiovascular events. Bold values indicate statistical significance, *P *< 0.05.

We repeated the MACCE Cox regression analysis using the subgroups of positive SE tests. In the multivariable analysis, the SE result was found to be the only predictor of MACCE again (*P* = 0.042). Both subgroups of apical ischaemia and the remaining positive SE tests had worse outcome with regards to MACCE when compared to patients with a negative SE (HR: 3.68; 95% CI: 1.06–12.78; *P* = 0.041 and HR: 3.72; 95% CI: 1.25–11.11, *P* = 0.019, respectively).

## Discussion

The findings of our study are (a) the patients who have a positive SE have a 3.7 times higher risk of MACCE compared to patients who have a negative SE. That risk is independent of other cardiovascular risk factors and remains the only predictor of MACCE after adjustment for all variables, (b) the patients with a negative SE are in a very low risk of MACCE (1%) and cardiac death (0%) within a year after the SE test and (c) the subgroup of patients with limited apical ischaemia have worse outcome compared to patients with negative SE but similar outcome compared to patients with inducible ischaemia in other segments. However, the prevalence of coronary artery disease and the need for revascularisation are much lower in the limited apical ischaemia subgroup compared to the other positive group.

Marwick and coworkers (11) demonstrated that the presence of ischaemia in SE is predictive of cardiac events including cardiac death, myocardial infarction, unstable angina and late revascularisation. They report that inducible ischaemia has an incremental predictive value added to other exercise data like exercise capacity, peak heart rate and blood pressure. We did not investigate the role of exercise parameters like METS and total exercise time as there were very few events in the ESE subgroup, making the analysis unreliable. Sicari and coworkers (10) included clinical parameters like diabetes, hyperlipidaemia and hypertension and found that the extent of ischaemia was an independent predictor of cardiac death along with age and previous myocardial infarction. We included the same cardiovascular risk factors as well as smoking history and previous history of CAD and found that only the stress echo result was predictive of MACCE. This is irrespective of the treatment provided (medical management vs revascularisation). The role of revascularisation has been assessed in several trials (15, 16, 17). However, our findings are important in that they demonstrate that immediately after a stress echo test, a prediction can be provided in terms of MACCE.

On the other hand, the SE result is not a predictor of all-cause mortality in our study. Sicari and coworkers (10) reported that the positive SE was significantly related to mortality in univariable analysis. The population in their study was younger (mean age 59 years vs 61 years in our study) and the prevalence of diabetes (14% vs 25.6%), hypertension (29% vs 63.8%) and hypercholesterolaemia (40% vs 59.5%) was lower. Therefore, we have dealt with a population that has a higher rate of comorbidities. The proportion of positive SE tests was similar (39% vs 41.7%) in both studies. In our population, 76% of recorded deaths were non-cardiac, whereas in the study by Sicari and coworkers (10), the non-cardiac deaths accounted for 52.1%. Recently, Cortigiani and coworkers (18) reported that inducible ischaemia in SE is correlated to all-cause mortality in a very large cohort of diabetic and non-diabetic patients. They were not able to separate cardiac deaths and hence reported all-cause mortality.

In our study, the patients who had limited apical ischaemia did not have a more favourable outcome in terms of MACCE compared to those who had inducible ischaemia in other segments. However, they were less likely to have more than mild CAD when investigated with ICA. Subsequently, the overall need for revascularisation was significantly lower (Table 5). From the 58 patients who underwent ICA, 36 (62.1%) did not have evidence of CAD or it was graded as only mild. Therefore, these patients demonstrated evidence of ischaemia with regional wall motion abnormalities only in the apical region at peak stress, but they did not have more than mild atherosclerosis in the large epicardial vessels. From the 22 (37.9%) patients who had moderate or severe coronary artery disease, twelve were revascularised.

**Figure 3:**
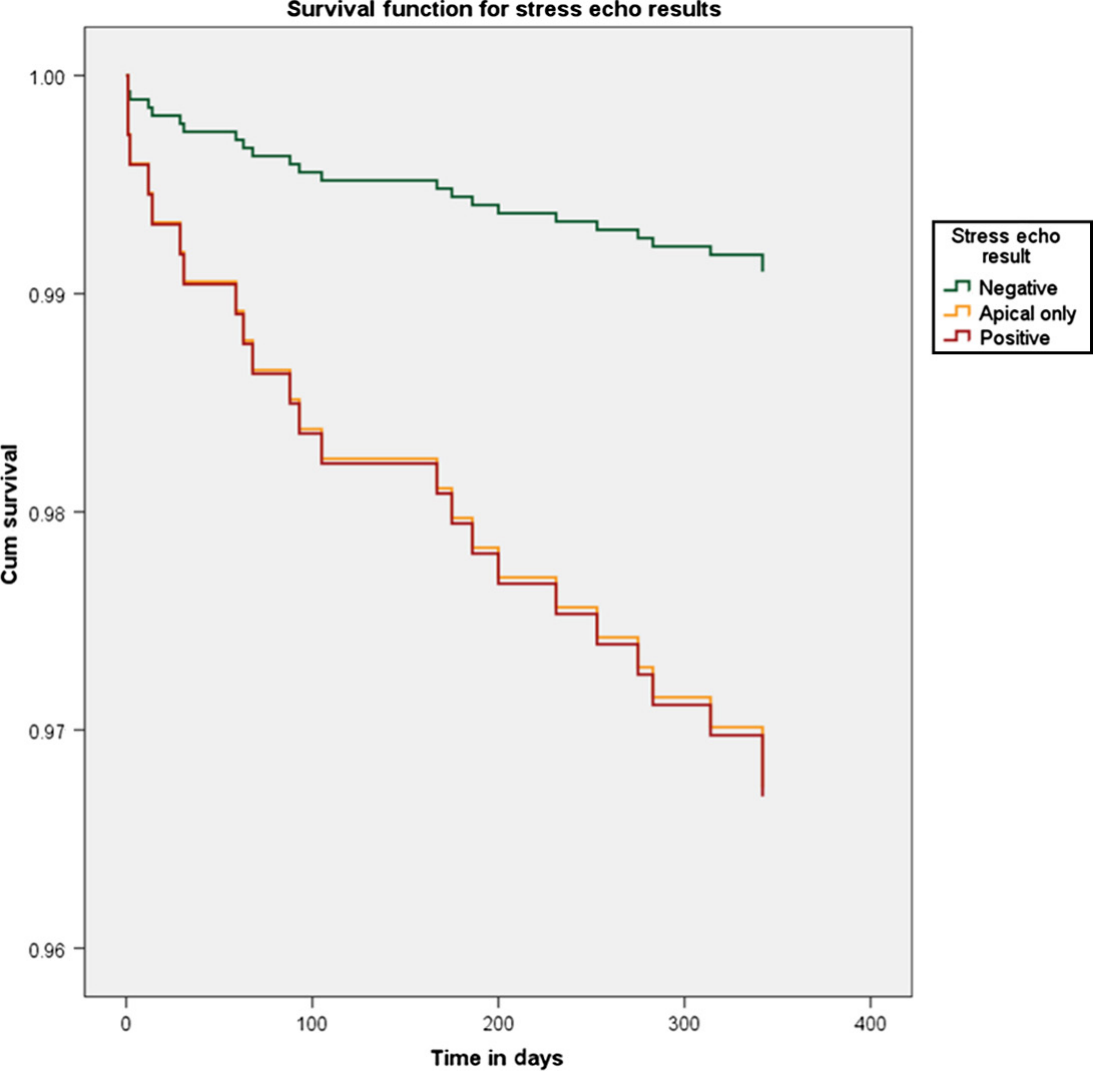
Cox regression survival curves for MACCE according to stress echo result subgroups.

These observations in the subgroup of patients with limited apical ischaemia in SE is a novel finding in the literature, and the low prevalence of more than mild coronary artery disease in this group of patients could be explained in a way that the induced limited apical ischaemia may suggest disease in small apical branches or microvascular disease. However, it is very interesting that the risk of MACCE is very similar to that of the group of patients who had inducible ischaemia in other segments (Fig. 3). Previously published data (19) pooled from 3 Thrombolysis in Myocardial Infarction (TIMI) trials reported 12.1% rate of MACCE in 1-year follow-up of patients who presented with NSTEMI and had normal or near normal findings on coronary angiography. With regards to stress echocardiography, it is not uncommon for clinicians to underestimate the clinical significance of ischaemia limited to apical segments.

Panza and coworkers (20) studied 70 patients who presented with typical anginal chest pain with normal or near normal coronary arteries (<30% epicardial coronary stenosis) in ICA. They used dobutamine stress echo and transoesophageal echocardiography (TOE) to assess inducible ischaemia. They did not show correlation of symptoms with regional wall motion abnormalities on SE. They utilised a quantitative analysis to assess myocardial response based on systolic thickening. However, the use of TOE may have limited ability to visualise the apex adequately. In our study, we used a qualitative method to assess regional wall motion abnormalities and we considered the possibility of false-positive SE tests. Therefore, a second expert operator who was blinded to initial report re-assessed the studies. For all cases that were grouped as ‘limited apical ischaemia’, there was an agreement between the two operators.

Our study includes a high proportion of dobutamine SEs as opposed to exercise SEs. The reason for that is that the accuracy of stress echocardiography is very strongly related to the image quality. We try to keep high standards of image quality in SEs performed in the department, and it is our personal experience that the images obtained during dobutamine stress echocardiography are of higher quality compared to exercise echocardiography irrespective of contrast use.

This is a single centre study. We did not record and we did not take into account the degree of medical treatment provided for primary or secondary prevention of CAD which is well-known to influence the outcome of these patients (1). We took account of the cerebrovascular accidents in the follow-up events though other large studies have focused on cardiac death, cardiac events and revascularisation only (11, 18). However, there is strong link between CAD and cerebrovascular disease both clinically and genetically (21, 22), and we consider that they should be investigated in common. Indeed 21% of the events recorded in the follow-up period were cerebrovascular accidents. Our patients were not investigated with Doppler coronary flow reserve or other validated methods to confirm microvascular disease (23, 24); therefore, certain correlations between limited apical ischaemia and microvascular disease in patients with normal coronary arteries cannot be made. We also did not assess myocardial perfusion in patients who received ultrasound contrast. This has proven useful in previous studies (25) and might have provided further insight, especially in patients with positive SE and normal or nearly normal coronary arteries in CA. Despite having agreement by two independent experienced operators for the ‘limited apical ischaemia’ SEs, there may still be a few false-positive cases in this group due to technical reasons, in particular foreshortening of the apex. Also the coronary angiography was reported by an experienced interventional cardiologist, but we did not use a quantitative method. Finally, we did not include data about renal function, which is well-known to be related with cardiovascular events and mortality, but this is consistent with previous registries.

## Conclusions

This is a contemporary study of 880 patients who underwent SE in a high-volume tertiary centre. The patients who had a positive SE test were found to have a 3.7 times higher risk of MACCE compared to those who had negative SE test. A negative SE warrants a very good prognosis within a year after the SE test. The all-cause mortality was similar in both groups as there was a high percentage of non-cardiac deaths. The subgroup analysis of positive SE tests did not show difference in outcome between limited apical ischaemia and other positive tests. However, the patients with limited apical ischaemia had significantly lower incidence of obstructive CAD when tested with invasive coronary angiography. Both subgroups of positive SE test had worse outcome compared to the patients with a negative SE test. The unexpected finding of relatively poor outcome, despite low incidence of obstructive CAD in patients with limited apical ischaemia is important, needs further investigation and suggests that the presumed presence of microvascular disease is prognostically important. CIAO, the registry arm of the Ischaemia trial (https://clinicaltrials.gov/ct2/show/Nbib1471522), is looking at the medical therapy of patients with positive stress echo and non-obstructive CAD and may shed more light on this in the near future.

## Declaration of interest

The authors declare that there is no conflict of interest that could be perceived as prejudicing the impartiality of the research reported.

## Funding

This research did not receive any specific grant from any funding agency in the public, commercial or not-for-profit sector.
